# Association of Occlusal Support With All-Cause 10-Year Mortality in Healthy, Community-Dwelling, 80-Year-Old Adults

**DOI:** 10.1016/j.identj.2025.103909

**Published:** 2025-09-23

**Authors:** Kohei Tamura, Kaname Nohno, Hiroshi Ogawa

**Affiliations:** a Division of Preventive Dentistry, Department of Oral Health Science, Faculty of Dentistry & Graduate School of Medical and Dental Sciences, Niigata University, Niigata, Japan; b Division of Oral Science for Health Promotion, Department of Oral Health and Welfare, Faculty of Dentistry & Graduate School of Medical and Dental Sciences, Niigata University, Niigata, Japan

**Keywords:** Dental occlusion, Mortality, Prognosis, Aged, Epidemiology

## Abstract

**Objectives:**

To evaluate the association between 10-year mortality and occlusal support according to the Eichner index (EI) in healthy community-dwelling 80-year-old adults.

**Methods:**

The participants were 360 community-dwelling 80-year-old adults. Follow-up surveys were conducted from June 2008 to June 2018, and all-cause mortality was confirmed by family members or caregivers. Those who (1) lost contact, (2) refused to participate, or (3) whose death dates were unknown were “censored” in the month when they were last confirmed to be alive. At baseline, dental and periodontal examinations, stimulated salivary flow measurements (gum method), blood tests (serum albumin), and self-administered questionnaire surveys (smoking and drinking habits, exercise habits, subjective health, years of education, and medical history) were conducted. From oral examination forms, occlusal support was classified into EI classes A/B and C, and the periodontal inflamed surface area was calculated. The Kaplan–Meier method and log-rank test were used for the survival analysis, and Cox regression proportional hazards models were used for multivariate analysis to calculate the hazard ratio (HR).

**Results:**

The analysis included a total of 297 participants, of which 203 had EI class A/B and 94 had class C at baseline, and their 10-year cumulative survival rates were 79.8% and 66.0%, respectively (*P = .*038). In the multivariate Cox proportional hazards model, men and EI class C were significant independent risk for all-cause mortality. HRs were 1.88 (95% confidence interval [CI] 1.08-3.36) for EI class C and 2.28 ([CI] 1.23-4.26) for men. In the strata analysis by sex, EI class C was a significant independent risk factor with an HR of 4.17 (95% CI 4.17-11.79) only in women.

**Conclusions:**

In healthy community-dwelling 80-year-old adults, Eichner class C, i.e., loss of occlusal support, was an independent risk for all-cause mortality, especially in women.

## Introduction

Mastication is an important factor in overall health and longevity.^1^^,^^2^ It involves coordinated movements of the lips, cheeks, tongue, masticatory muscles, mandible, and neck, all functioning together—based on a stable bite with teeth or dentures—to grasp, bite, crush, grind, and swallow food mixed with saliva.^3^ Therefore, both the number of teeth and adequate occlusal support are essential for effective mastication. Previous studies have reported that a decline in occlusal support decreases both mastication satisfaction and efficiency.^4^^,^^5^ This deterioration is primarily due to the progression of periodontal diseases and dental caries, which have also been linked to inadequate nutrient intake and impaired motor function.^6^^,^^7^

Frailty has recently gained attention as a factor influencing life expectancy in older adults, with meta-analyses identifying it as a significant risk factor for mortality.^8^^,^^9^ Similarly, oral-related factors—including tooth loss, xerostomia, poor oral hygiene, and impaired swallowing function—have also been linked to increased mortality risk.^10^, ^11^, ^12^, ^13^, ^14^, ^15^ Although some studies have explored associations between the number of functional teeth or occlusal force and mortality,^16^, ^17^, ^18^ the populations studied have varied widely in age range and health status. According to our research, no study has explicitly focused on healthy 80-year-olds—a demographic projected to grow rapidly. According to the Japanese Ministry of Health, Labor, and Welfare, the average life expectancy in Japan as of 2022 was 81.05 years for men and 87.09 years for women. Regarding oral health, the 8020 Campaign reported that 51.6% of people aged 75-84 had at least 20 teeth as of 2022. These findings highlight the growing importance of tooth preservation and the maintenance of oral function in older adults. To support a good long-term prognosis, dental professionals must identify effective strategies for maintaining oral health for adults aged ≥80 years. Regarding the relationship between occlusion and mortality, Iinuma et al.^17^ found that maximum bite force was independently associated with all-cause mortality. Similarly, the number of functional teeth has been reported to be a stronger predictor of all-cause mortality than the total number of teeth.^18^ We hypothesized that the Eichner Index (EI) may serve as a proxy for these indicators.

This study, as part of a 25-year cohort study of older adults in Niigata City, tested the hypothesis that loss of occlusal support is a risk factor for mortality. Specifically, we examined the association between 10-year mortality and occlusal support—measured by the EI—in healthy, community-dwelling 80-year-olds without frailty.

## Methods

### Study design and population

This longitudinal cohort study began in 1998. Survey request forms explaining the purpose of the study were mailed to 4542 registered 70-year-old residents of Niigata City, and 3,695 (81.4%) agreed to participate. From these respondents, 600 individuals (306 men and 294 women) were randomly selected using computer‐based sampling to ensure an approximately equal distribution of each sex and a manageable sample size for clinical examinations. Assessments were conducted at various community centers and gymnasiums in the city. None of the participants were hospitalized or institutionalized at the time. Annual follow-up examinations continued through 2018. The baseline for this analysis was set in June 2008, when 360 of the participants had reached the age of 80. Participants were excluded if they (1) were frail or could not be assessed for frailty, (2) died in June 2008, or (3) had missing oral health data. After exclusion, 297 participants (155 men and 142 women) remained. A post hoc power analysis was conducted using G*Power (ver. 3.1.9.7) to assess the difference in 10-year mortality rates between the EI class A/B group and the class C group.

**Fig. 1:**
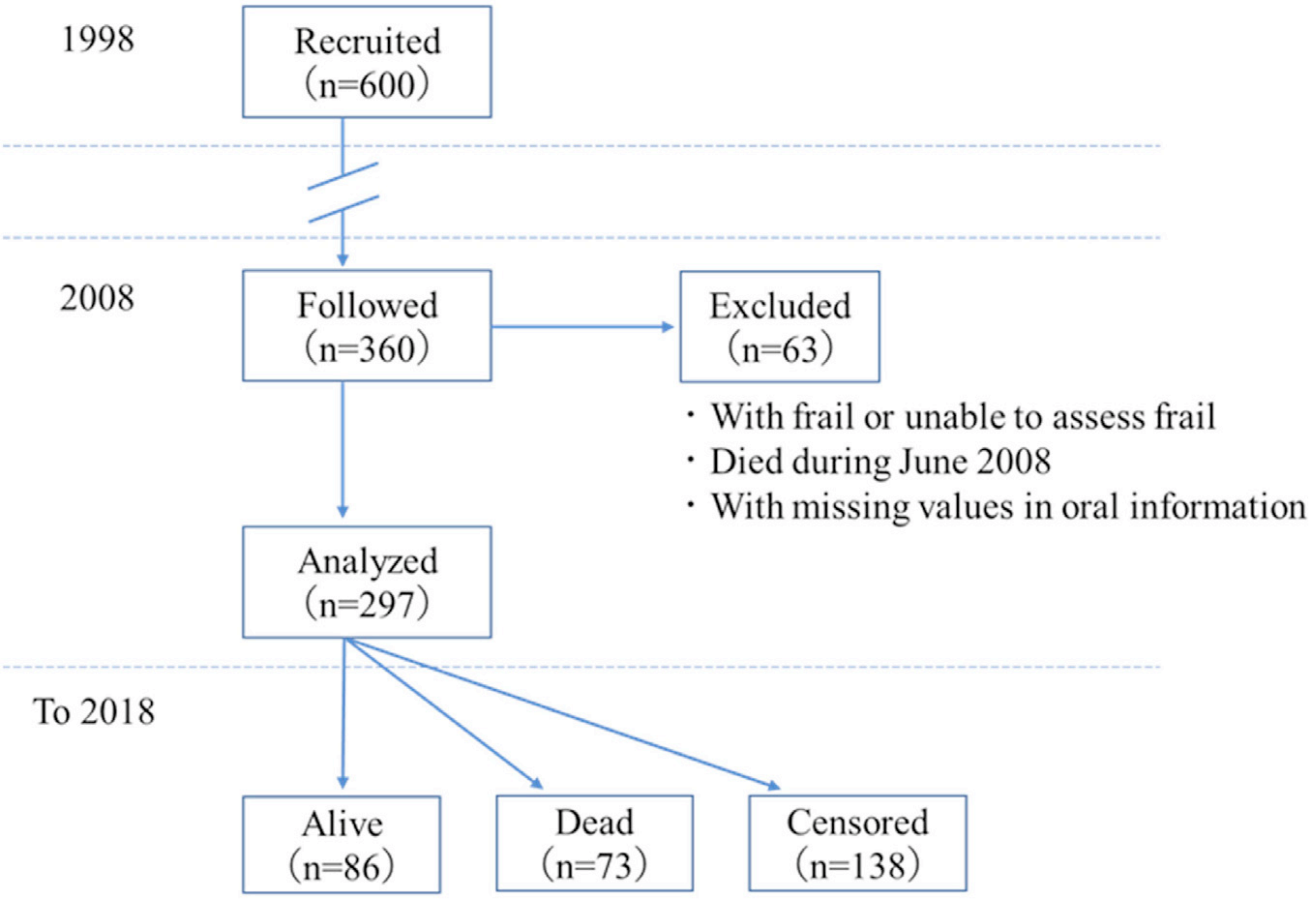
Participant selection process for the analyses in this study.

Survival status and the dates of death were confirmed annually from 2008 to 2018 through follow-up telephone calls. Participants lost to follow-up were censored on the date they were last seen or contacted. The participants’ selection process for the longitudinal analysis is shown in Figure 1.

This study was conducted in accordance with the Declaration of Helsinki and approved by the Ethics Committee of the Niigata University Faculty of Dentistry (Approval no. 2015-5001).

### Baseline examination

Four calibrated dentists assessed each participant’s number of remaining teeth, denture status, and periodontal status. The remaining teeth were defined as erupted permanent teeth, excluding residual roots. Periodontal assessment included probing pocket depth and bleeding on probing, recorded at 6 sites per tooth. Stimulated whole saliva was collected based on a previously published protocol.^19^ The cutoff value for decreased stimulated salivary flow was set at 1.0 mL/min based on the cutoff value for Sjögren’s syndrome.^20^ Participants completed a questionnaire on socioeconomic status (education level), health behaviors (smoking, alcohol consumption, and exercise), and medical history, including hypertension, diabetes, and cardiovascular diseases (CVDs). Height and weight were measured to calculate the body mass index (BMI), with low body weight and obesity defined as a BMI <18.5 and ≥25.0 kg/m^2^, respectively. Fasting blood samples were collected to measure serum albumin levels; concentrations ≤4.1 mg/dL (median) were classified as low. According to the criteria by Freid et al.,^21^ participants were classified as frail if they exhibited 3 or more of the following 5 symptoms: unintentional weight loss (≥2 kg over 6 months), muscle weakness (grip strength <28 kg for men and <18 kg for women), fatigue (feeling tired without reason in the past 2 weeks), reduced walking speed (normal walking speed <1.0 m/sec), and low physical activity (no regular exercise). Nutritional intake was assessed using a brief self-administered diet history questionnaire (BDHQ).^22^ Nutrient intakes were adjusted per 1000 kcal of energy intake.

Occlusal condition was evaluated using the EI based on dental examination records. EI was determined by assessing the contact status of the natural teeth—excluding residual roots—between the maxilla and mandible. Fixed prosthetic appliances, including pontics from fixed partial dentures and implant-supported crowns, were considered in the occlusal status assessment. EI classifications were defined as follows: class A (represents occlusal support in all quadrants), class B (support loss in 1-3 quadrants or anterior contact only), and class C (no occlusal support). Participants were grouped as having occlusal support (classes A or B) or lacking it (class C). The periodontal epithelium surface area (PESA, mm^2^) and periodontal inflamed surface area (PISA), derived from the PESA, were calculated using freely available Excel toolsets (https://www.parsprototo.info). A PISA value >21.3 mm^2^, the median of the study population, was defined as high.

### Statistical methods

All statistical analyses were conducted using IBM SPSS Statistics for Windows, version 28 (IBM Corp., Armonk, NY, USA). A significance level of *P < .*05 was applied. In 2008, participant characteristics—including sex, oral health status, general health, educational background, lifestyle habits, and comorbidities—were compared between EI classes A/B and class C using the chi-square test. For survival analysis, the survival period was calculated in months from the baseline examination (June 2008) to the date of death or censoring (last follow-up or telephone contact). Cumulative survival rates were estimated using the Kaplan–Meier method. Unadjusted survival curves were compared between men and women and among the 3 EI classes using the log-rank test. To illustrate the significant impact of frailty on life prognosis, cumulative survival rates were also plotted for the entire study population, including participants with frailty. Survival curves across EI classes within this subgroup were compared using the log-rank test, and the unadjusted survival curves between the 3 EI classes were compared using the log-rank test. Univariable and multivariable Cox proportional hazards regression models were used to assess the crude and adjusted effects of EI classification in 2008 on mortality.

## Results

Table 1 presents the baseline characteristics of the participants in 2008. Significant differences were observed between EI classes A/B and class C in the mean survival time (*P = .*038), the number of teeth (*P < .*001), PISA (*P < .*001), denture use (*P < .*001), regular exercise (*P = .*021), and smoking status (*P = .*025).

**Table 1:** Baseline characteristics stratified by the Eichner index.

Characteristics in 2008	Category	Class A/B	Class C	*P*-value*
		n = 203	n = 94	
Mean survival time (months)		108.51±1.86	103.24±2.98	.038
Sex, n (%)				
	Women	99 (48.8)	43 (45.7)	N.S.
	Men	104 (51.2)	51 (54.3)	
The number of teeth, n (%)				
	≥20	129 (100)	0 (0)	<.001
	10-19	64 (84.2)	12 (15.8)	
	0-9	10 (10.9)	82 (89.1)	
PISA, n (%)				
	<21.38 mm^2^	78 (38.4)	71 (75.5)	<.001
	≥21.38 mm^2^	125 (61.6)	23 (24.5)	
Stimulated salivary flow rate, n (%)				
	>1.0mL/1m	147 (72.4)	61 (64.9)	N.S.
	≤1.0mL/1m	56 (27.6)	33 (35.1)	
Denture use, n (%)				
	Yes	97 (47.8)	91 (96.8)	<.001
	No	106 (52.2)	3 (3.2)	
Serum albumin, n (%)				
	>4.1g/dl	83 (41.9)	32 (35.6)	N.S.
	≤4.1g/dl	115 (58.1)	58 (64.4)	
BMI (kg/㎡), n (%)				
	≥25	28 (13.8)	19 (20.2)	N.S.
	<25	156 (76.8)	62 (66.0)	
	<18.5	19 (9.4)	13 (13.8)	
Subjective health, n (%)				
	Healthy	151 (74.8)	74 (79.6)	N.S.
	Unhealthy	51 (25.2)	19 (20.4)	
Years of education, n (%)				
	≥10 years	122 (60.7)	52 (55.3)	N.S.
	≤9 years	79 (39.3)	42 (44.7)	
Regular exercise, n (%)				
	Yes	124 (61.1)	44 (46.8)	.021
	No	79 (38.9)	50 (53.2)	
Smoking status, n (%)				
	Never	131 (65.8)	51 (54.3)	.025
	Former	55 (27.6)	28 (29.8)	
	Current	13 (6.5)	15 (16.0)	
Alcohol consumption, n (%)				
	No	111 (55.8)	52 (55.3)	N.S.
	Not every day	49 (24.6)	18 (19.1)	
	Everyday	39 (19.6)	24 (25.5)	
Heart disease, n (%)				
	No	186 (92.5)	82 (87.2)	N.S.
	Yes	15 (7.5)	12 (12.8)	
Lifestyle disease, n (%)				
	No	112 (55.7)	56 (59.6)	N.S.
	Yes	89 (44.3)	38 (40.4)	

⁎ The tests for differences between EI class A/B and C are the χ2 test for categorical variables and log-rank test for survival months.

Table 2 reveals the difference between the number of deaths and survivors during the 2018 follow-up period. Among participants with EI classes A/B, a significant difference was found based on sex (*P = .*001), PISA (*P = .*013), and subjective health (*P = .*028). In contrast, for those in EI class C, only subjective health showed a significant difference between non-survivors and survivors (*P = .*004).

**Table 2:** Ratio of deaths and survivors*.

Characteristics in 2008	Category	Class A/B			Class C			
		Death	Survivor	*p*-value†	Death	Survivor	*p*-value†	
		n (%)	n (%)		n (%)	n (%)		
Sex								
	Women	9 (20.9)	34 (79.1)	.001	12 (48.0)	13 (52.0)	N.S.	
	Men	32 (52.5)	29 (47.5)		20 (66.7)	10 (33.3)		
The number of teeth								
	≥20	26 (38.2)	42 (61.8)	N.S.	-	-	N.S.	
	10−19	12 (40.0)	18 (60.0)		6 (75.0)	2 (25.0)		
	0−9	3 (50.0)	3 (50.0)		26 (55.3)	21 (44.7)		
PISA								
	<21.38 mm^2^	11 (25.6)	32 (74.4)	.013	23 (56.1)	18 (43.9)	N.S.	
	≥21.38 mm^2^	30 (49.2)	31 (50.8)		9 (64.3)	5 (35.7)		
Stimulated salivary flow								
	>1.0mL/1m	32 (40.0)	48 (60.0)	N.S.	21 (60.0)	14 (40.0)	N.S.	
	≤1.0mL/1m	9 (37.5)	15 (62.5)		11 (55.0)	9 (45.0)		
Serum albumin								
	>4.1g/dl	13 (28.9)	32 (71.1)	N.S.	11 (52.4)	10 (47.6)	N.S.	
	≤4.1g/dl	26 (45.6)	31 (54.4)		19 (59.4)	13 (40.6)		
BMI (kg/㎡)								
	≥18.5	37 (39.4)	57 (60.6)	N.S.	27 (55.1)	22 (44.9)	N.S.	
	<18.5	4 (40.0)	6 (60.0)		5 (83.3)	1 (16.7)		
Subjective health								
	Healthy	26 (33.8)	51 (66.2)	.028	23 (50.0)	23 (50.0)	.004	
	Unhealthy	15 (57.7)	11 (42.3)		9 (100)	0 (0)		
Years of education								
	≥10 years	26 (38.2)	42 (61.8)	N.S.	15 (50.0)	15 (50.0)	N.S.	
	≤9 years	15 (41.7)	21 (58.3)		17 (68.0)	8 (32.0)		
Regular exercise								
	Yes	24 (34.8)	45 (65.2)	N.S.	15 (55.6)	12 (44.4)	N.S.	
	No	17 (48.6)	18 (51.4)		17 (60.7)	11 (39.3)		
Smoking status								
	Never/Former	34 (37.4)	57 (62.6)	N.S.	24 (53.3)	21 (46.7)	N.S.	
	Current	6 (50.0)	6 (50.0)		8 (80.0)	2 (20.0)		
Alcohol consumption								
	No	19 (36.5)	33 (63.5)	N.S.	15 (46.9)	17 (53.1)	N.S.	
	Yes	21 (41.2)	30 (58.8)		17 (73.9)	6 (26.1)		
Heart disease								
	No	36 (37.1)	61 (62.9)	N.S.	27 (54.0)	23 (46.0)	N.S.	
	Yes	4 (66.7)	2 (33.3)		5 (100)	0 (0)		
Lifestyle disease								
	No	25 (44.6)	31 (55.4)	N.S.	17 (56.7)	13 (43.3)	N.S.	
	Yes	15 (31.9)	32 (68.1)		15 (60.0)	10 (40.0)		

⁎ Exclude subjects who were censored and list deaths and survival at the end of observation.

† The tests for differences between EI class A/B and C are the χ2 test for categorical variables.

**Fig. 2:**
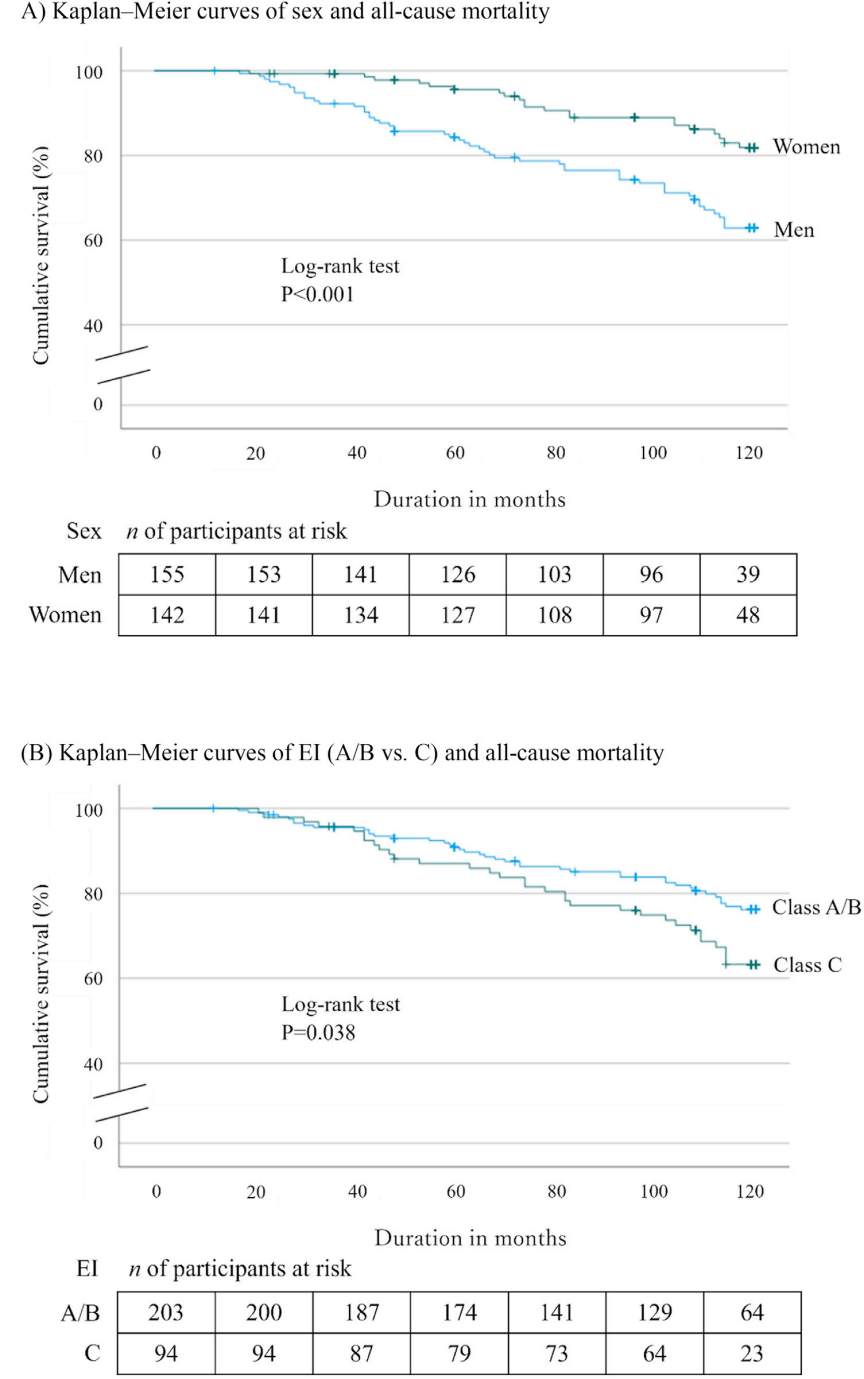
Cumulative survival rates for all-cause mortality plotted using the Kaplan–Meier method.

Kaplan–Meier survival curves for all-cause mortality were plotted by sex and EI (A/B vs. C) at baseline (Figure 2). Log-rank tests indicated significant differences between men and women (*P < .*001) (Figure 2A) and between EI classes A/B and C (*P = .*038) (Figure 2B).

Table 3 shows the Cox proportional hazards regression analyses of predictors associated with all-cause mortality. In the univariable models, EI class C was significantly associated with increased mortality (hazard ratio [HR], 1.62; 95% confidence interval [CI], 1.02-2.58). This association remained significant and became stronger after adjusting for confounders, including sex, serum albumin, BMI, smoking status, alcohol consumption, subjective health, education level, regular exercise, lifestyle disease, and heart disease (adjusted HR [aHR], 1.88; 95% CI, 1.08-3.26). Additionally, sex, subjective health, and smoking status were significantly associated with all-cause mortality in the univariate models. Among these, only sex remained significant after adjusting (HR, 2.47; 95% CI, 1.49-4.10; aHR, 2.28; 95% CI, 1.23-4.26).

**Table 3:** Crude and adjusted hazard ratios for mortality.

Predictor	Crude HR (95%CI)	Adjusted HR (95%CI)
Sex (women vs. men)	**2.47 (1.49-4.10)**	**2.28 (1.23-4.26)**
EI (class A/B vs. class C)	**1.62 (1.02-2.58)**	**1.88 (1.08-3.26)**
The number of teeth (≥10 vs. 0-9)	1.32 (0.83-2.11)	**-**
(≥20 vs. 0-19)	1.32 (0.81-2.13)	**-**
PISA (<21.38 mm^2^ vs. ≥21.38 mm^2^)	1.25 (0.79-1.98)	1.60 (0.91-2.79)
Stimulated salivary flow rate (≤3.0mL/3m vs. >3.0mL/3m)	1.17 (0.70-1.96)	1.08 (0.62-1.89)
Serum albumin (4.1g/dl< vs. ≦4.1g/dl)	1.32 (0.80-2.16)	0.96 (0.57-1.63)
BMI (<18.5kg/㎡ vs. ≥18.5kg/㎡)	1.27 (0.63-2.56)	1.01 (0.47-2.21)
Subjective health (healthy vs. unhealthy)	**1.95 (1.20-3.18)**	1.59 (0.90-2.83)
Years of education (≥10 years vs. ≤9 years)	1.17 (0.74-1.86)	1.20 (0.73-1.96)
Regular exercise (yes vs. no)	1.23 (0.78-1.94)	1.31 (0.80-2.14)
Current smoker (vs. previous smoker/ nonsmoker)	**2.52 (1.41-4.53)**	1.64 (0.85-3.16)
Current drinker (vs. nondrinker)	1.37 (0.86-2.18)	0.91 (0.54-1.56)
Heart disease (yes vs. no)	1.57 (0.78-3.16)	1.01 (0.43-2.39)
Lifestyle disease (yes vs. no)	0.93 (0.58-1.49)	0.89 (0.54-1.47)

HR, hazard ratio; CI, confidence interval.

Bold text indicates statistically significant associations (*P < .*05).

Table 4 presents sex-stratified Cox regression analyses of predictors of all-cause mortality. For women, EI class C was strongly associated with mortality (aHR, 4.17; 95% CI, 1.47-11.79). Conversely, the association in men was weaker and did not reach statistical significance.

**Table 4:** Crude and adjusted hazard ratios for the Eichner index in 2008 stratified by sex.

EI in 2008	Crude HR (95%CI)	Adjusted HR* (95%CI)
Men		
Class A/B	Reference	Reference
Class C	1.27 (0.73-2.23)	1.19 (0.58-2.47)
Women		
Class A/B	Reference	Reference
Class C	**2.79 (1.18-6.63)**	**4.17 (1.47-11.79)**

⁎ Adjusted for PISA, stimulated salivary flow rate, smoking status, serum albumin, BMI, regular exercise, subjective health, drinking, education, heart disease and lifestyle disease. HR, hazard ratio; CI, confidence interval. Bold text indicates statistically significant associations (*P < .*05).

As shown in Table 5, intake of vitamins B2, B9, and C, as well as omega-6 fatty acids, was significantly higher in participants with EI class A/B than in those with class C.

**Table 5:** Total energy and nutrient intake(adjusted value) per day by Eichner index.

Nutrients	EI				*p*-value*
	Class A/B		Class C		
	Mean	±SD	Mean	±SD	
Total energy (kcal)	2067.12	(637.10)	2157.29	(697.68)	N.S.
Protein (g)	16.21	(2.96)	16.06	(3.30)	N.S.
Lipid (g)	29.55	(4.97)	28.55	(4.91)	N.S.
Carbohydrate (g)	51.66	(6.89)	51.83	(7.21)	N.S.
Vitamin B1 (g)	0.47	(0.89)	0.46	(0.86)	N.S.
Vitamin B2 (g)	0.81	(0.17)	0.77	(0.15)	.035
Vitamin B3 (g)	9.72	(2.48)	9.77	(2.60)	N.S.
Vitamin B5 (g)	3.90	(0.65)	3.76	(0.57)	N.S.
Vitamin B9 (g)	242.88	(63.86)	224.73	(50.44)	.016
Vitamin C (g)	93.66	(30.10)	86.66	(25.29)	.039
Vitamin D (g)	10.67	(5.54)	10.69	(7.03)	N.S.
Vitamin K (g)	229.29	(93.31)	210.43	(85.97)	N.S.
Omega-3 fatty acid (g)	1.99	(0.52)	1.89	(0.58)	N.S.
Omega-6 fatty acid (g)	6.95	(1.37)	6.6	(1.41)	.047

⁎ The tests for differences between EI class A/B and C are the t test for nutrient variables.

**Fig. 3:**
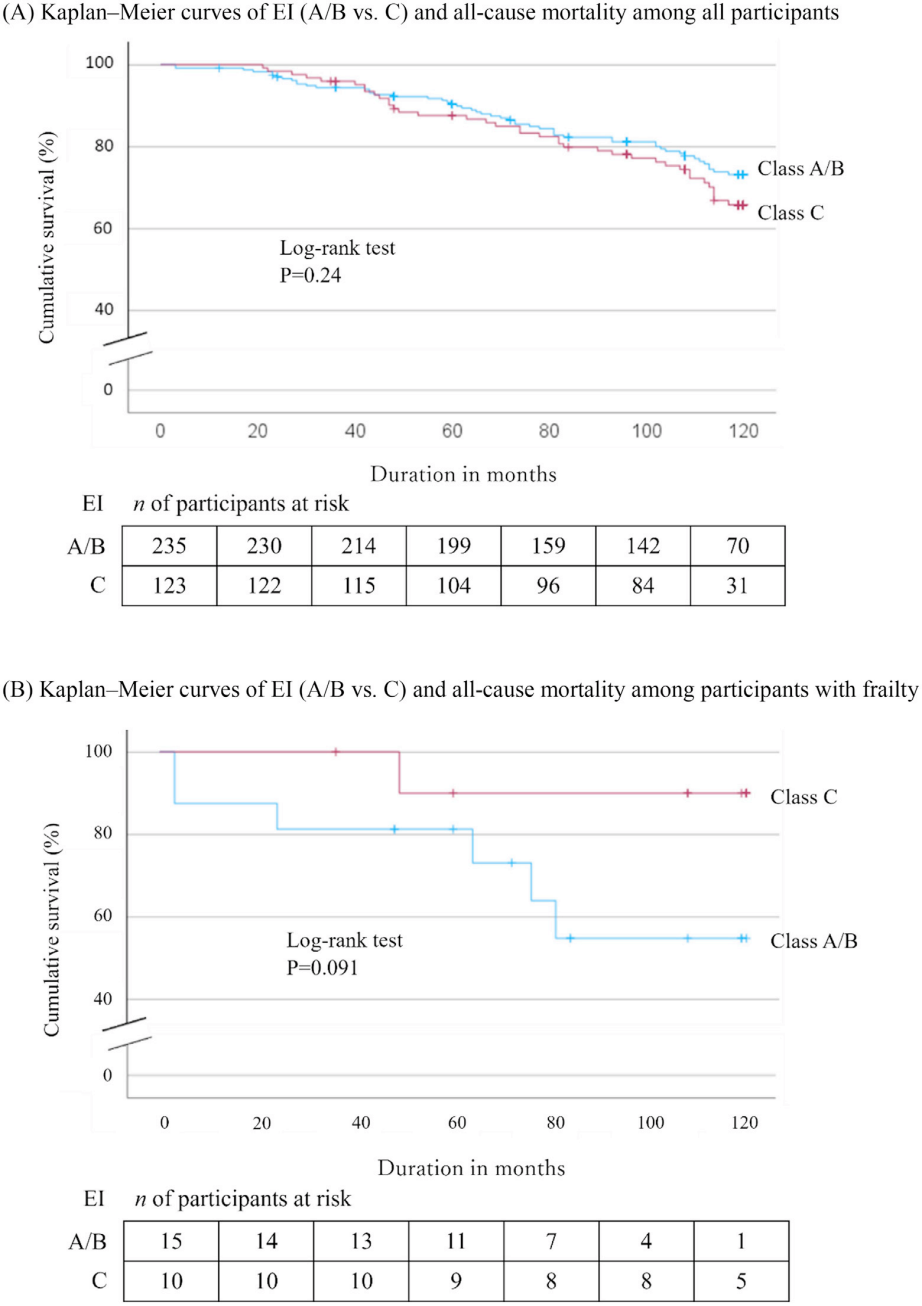
Cumulative survival rates for all-cause mortality plotted using the Kaplan–Meier method.

Analysis of participants with frailty showed no significant differences in survival between the EI classes, as indicated by the log-rank test for both the overall study population and the frail group (Figure 3).

## Discussion

This 10-year prospective cohort study found that the loss of occlusal support—including cases with fixed prosthetic appliances—was a significant predictor of mortality in healthy, community-dwelling 80-year-old adults. Survival rates also differed significantly between men and women. In the stratified analysis, the association between occlusal support loss and mortality remained independently significant only among women. While 1 previous study reported a relationship between the change in occlusal support over a 5-year period from the age of 75 and subsequent life expectancy,^23^ no study has reported occlusal support at a single time point as a risk factor for mortality. To the best of our knowledge, this is the first study to show this relationship.

No significant difference in survival was observed between EI classes A/B and C in the overall study population or the frailty subgroup, as indicated by the log-rank test. However, a significant difference was found in the non-frail group when stratified by frailty status. This may be explained by the strong influence of frailty as a risk factor for mortality, as shown in previous studies, and by the exclusion of the frail group from the main analysis.^8^^,^^9^

In this study, participants with EI classes A/B and C differed significantly in PISA, regular exercise, and smoking status. Our results are consistent with prior research linking smoking and tooth loss.^24^^,^^25^ Hosoda et al. also reported an association between certain lifestyle habits and tooth loss.^26^ However, few studies have specifically examined the relationship between exercise habits and tooth loss. Therefore, further research is needed to clarify this association and identify potential mediating factors.

The difference in mortality rates between EI classes A/B and C may be attributed to several factors. First, the loss of occlusal support likely contributed to reduced masticatory function. Previous studies have reported that both masticatory efficiency and bite force decrease progressively from EI class A to B to and C.^17^^,^^27^^,^^28^ Occlusal support from remaining teeth has been identified as the most important determinant of the bite force, with odds ratios for low bite force (≤238 N) of 3.6 in EI class B and 19.4 in class C, compared to class A.^29^ Although masticatory efficiency and bite force were not directly measured in this study, prior evidence suggests these likely declined with the loss of occlusal support. Consequently, reduced masticatory function may have negatively impacted nutritional status. Several studies have also reported an association between occlusion and nutritional outcomes. Appollonio et al.^30^ reported no significant differences in the intake of macronutrients—protein, fat, and carbohydrate—between people with maintained and disrupted occlusions. However, vitamin intake, including folic acid, was lower in the occlusion-disrupted group. They also indicated that the denture-wearing group within the occlusion-disrupted group had nutritional intake status levels similar to those of the group with maintained occlusion. Another study proposed that loss of occlusal contact with natural molar teeth decreases the intake of vitamins and dietary fibers,^31^ and while individuals with low bite force consume fewer green and yellow vegetables and seafood, resulting in a lower intake of antioxidant vitamins and dietary fibers.^32^ In this study, participants in EI class C had significantly lower intakes of essential nutrients, including vitamins B2, B9, and C. Deficiencies in these vitamins are associated with impaired immune function and increased mortality risk. In a 24-year follow-up study of middle-aged and older adults in the United States, Pandy et al. found that higher intakes of β-carotene (a vitamin A precursor) and vitamin C were independently associated with reduced all-cause mortality.^33^ A large-scale Chinese study involving 495,332 middle-aged and older adults found that higher total intakes of β-carotene and vitamin C were associated with reduced all-cause and CVD mortality.^34^

Furthermore, in this study, stratified analysis by gender revealed that the association between loss of occlusal support and increased mortality was particularly significant in women. Previous research suggests that the association between nutritional deficiencies and higher mortality is stronger in women, which may partly explain this gender difference. A Japanese study involving 58,730 participants aged 40-79 years revealed an association between vitamin C intake and CVD mortality only in women,^35^ and Kushi et al.^36^ also reported that dietary intake of vitamin E was inversely associated with coronary heart disease–related mortality in postmenopausal women. Vitamins A, C, and E—commonly referred to as antioxidant vitamins and primarily found in vegetables—are considered to be associated with systemic health, including immune function.^37^

Moreover, the loss of occlusal support may contribute more significantly to mortality risk in women through mechanisms not examined in this study, such as the progression of sarcopenia, increased psychosocial stress, and frailty. Older women were generally more susceptible to the progression of sarcopenia and frailty due to lower muscle mass and hormonal changes, such as decreased estrogen levels after menopause.^38^^,^^39^ A decline in masticatory function may hinder the consumption of protein-rich foods, potentially accelerating muscle mass loss and impaired physical function. Psychosocial factors may also contribute to gender differences in mortality risk. Declining oral health has been reported to be associated with depressive symptoms and social isolation.^40^^,^^41^ In older women, a strong correlation has been reported between mental health scores and subjective health status, suggesting that psychosocial stress may affect both physical health and quality of life.^42^ In our study, the loss of occlusal support may have increased the mortality risk in women, potentially mediated by psychosocial stress.

In this study, the 10-year survival rate was significantly lower in men than in women, and occlusal support loss was not associated with mortality among men. Menotti et al. reported that elevated comorbidities—including coronary heart disease—and smoking were independently associated with all-cause mortality in older men.^43^ They also found that the number of comorbidities—including coronary heart diseases, heart failure, CVDs, intermittent claudication, chronic obstructive pulmonary disease, diabetes, and cancer—was proportional to the HR for all-cause mortality.^44^ However, in our study, smoking was not associated with mortality in either sex, and the number of diseases was not evaluated.

Previous studies have identified periodontal disease and low salivary flow as potential risk factors for mortality.^45^^,^^46^ However, in the present study, no such associations were observed.

Several limitations should be noted. First, the 10-year survival rate from age 80 among our participants was 75.4% (excluding censored cases), whereas the corresponding rate for the Japanese population was 48.7%.^47^ This discrepancy likely reflects the survival bias, as participants were part of a larger survey and had been randomly selected in 1998 but were still alive as of 2008. Consequently, the generalizability of these findings to the broader population of the same age group should be interpreted with caution. Second, although a strict sample size was not predetermined, a post hoc power analysis indicated sufficient statistical power of 0.77 based on the 203 participants in the EI class A/B group (mortality rate: 20.2%) and 94 participants in the EI class C group (mortality rate: 34.0%). Among women, the power was 0.81, with 99 participants in the EI class A/B group (mortality rate: 9%) and 43 in class C (mortality rate: 28%), suggesting that the study was adequately powered to detect significant differences. Third, the analysis was limited to all-cause mortality, meaning that cause-specific mortality could not be assessed. This is an important consideration, as prior studies have reported associations between poor oral health and specific causes of death, such as CVDs and respiratory diseases. For example, in a Japanese cohort study of 4425 adults aged ≥65 years found that individuals with ≤19 teeth and eating difficulties had significantly higher multivariate-adjusted hazard ratios for CVD and respiratory disease mortality (1.83 and 1.85, respectively) compared to those with ≥20 teeth.^13^ A Swedish study reported a sevenfold increased risk of coronary heart disease–related mortality in participants with less than 10 teeth compared with those with over 25 teeth.^15^ Similarly, Kotronia et al. found that poor oral health was associated with CVDs and respiratory mortality.^14^

Additionally, the Eichner classification evaluates the structural state of occlusion but does not directly assess functional aspects, such as occlusal force or masticatory efficiency. However, previous studies have suggested that reduced occlusal support is linked to decreased occlusal force and masticatory function. Although denture use has been associated with improved prognosis,^48^ 97% of participants with occlusal support loss in this study wore dentures. Hence, we could not compare outcomes between denture users and non-users. Additionally, the questionnaire did not assess the presence, quality, or subjective experience of denture use.

Although the BDHQ used in the dietary survey has demonstrated a certain degree of validity for individuals aged ≥80, it has known limitations in estimating dietary intake,^49^ and its application in this age group should be treated with caution.

Occlusal support assessed using the EI, which accounts for fixed prosthetic appliances, may serve as a practical predictor of mortality in 80-year-olds, as it is easier to evaluate than other prognostic factors (e.g., nutrition status). Although the number of teeth was similarly easy to assess, no association with mortality was observed. Further research is needed to examine how the underlying causes of occlusal support loss and subsequent changes—such as restoration through prosthetic treatment or denture use—may influence mortality.

The present findings suggest a potential indirect causal pathway in which the loss of occlusal support leads to diminished masticatory function, which may impair nutrient intake and subsequently increase mortality risk. Although this mediation model—from impaired mastication to malnutrition, and ultimately, to elevated mortality—has not yet been empirically tested in this population, it offers a plausible explanation for the observed associations. Moreover, the potential link between occlusal support and increased psychological stress also requires further investigation. To clarify these mechanisms, future research should employ formal mediation analyses to examine how oral health conditions may influence longevity.

## Conclusions

In this cohort of older adults, EI class C was independently associated with all-cause mortality among Japanese community-dwelling 80-year-old women, even after adjustment for various health-related factors, including PISA, stimulated salivary flow rate, smoking status, serum albumin, BMI, regular exercise, subjective health status, alcohol consumption, educational attainment, heart disease, and other lifestyle-related conditions. These findings indicate that maintaining occlusal support may play an important role in promoting longevity by supporting masticatory function and adequate nutritional intake. Future studies should further examine the indirect pathway linking occlusal support loss to mortality.

## Authors contributions

KN and HO prepared and submitted the proposal for research grant and ethical approval. All authors participated in the data collection. KT performed a literature review, analyzed the data, and wrote and edited the manuscript. All authors reviewed the manuscript and revised it critically on intellectual content. KN supervised the conduct of this study, and HO was responsible for manuscript submission.

## Funding

This study was supported by JSPS KAKENHI grant 16K11884.

## Ethics statement

The study was conducted by the World Medical Association Declaration of Helsinki. All procedures were approved by the Ethics Committee of the Faculty of Dentistry, Niigata University (Approval no. 2015-5001). Written informed consent was obtained from all participants.

## Conflict of interest

None disclosed.

## References

[1] Nomura Y., Kakuta E., Okada A. (2020). Impact of the serum level of albumin and self-assessed chewing ability on mortality, QOL, and ADLs for community-dwelling older adults at the age of 85: a 15 year follow up study. Nutrients.

[2] Nomura Y., Kakuta E., Okada A. (2020). Effects of self-assessed chewing ability, tooth loss and serum albumin on mortality in 80-year-old individuals: a 20-year follow-up study. BMC Oral Health.

[3] van der Bilt A., Engelen L., Pereira L.J., van der Glas H.W., Abbink JH. (2006). Oral physiology and mastication. Physiol Behav.

[4] Dhingra S., Rajesh G., Rao A., Pai U.Y., Shenoy R., Pai M. (2017). Impact of occlusal support and perceived chewing ability on oral health-related quality of life among patients attending a private dental institution in India. J Indian Prosthodont Soc.

[5] Huang Y.F., Liu S.P., Muo C.H., Chang CT. (2021). The impact of occluding pairs on the chewing patterns among the elderly. J Dent.

[6] Mikami R., Komagamine Y., Aoyama N. (2023). Association between occlusal supports and nutritional status in older adults: a systematic review. J Dent Sci.

[7] Hatta K., Ikebe K., Mihara Y. (2019). Lack of posterior occlusal support predicts the reduction in walking speed in 80-year-old Japanese adults: a 3-year prospective cohort study with propensity score analysis by the SONIC study group. Gerodontology.

[8] Kojima G., Iliffe S., Walters K. (2018). Frailty index as a predictor of mortality: a systematic review and meta-analysis. Age Ageing.

[9] Romero-Ortuno R., Kenny RA. (2013). The frailty index in Europeans: association with age and mortality. Age Ageing.

[10] Saito M., Shimazaki Y., Nonoyama T., Tadokoro Y. (2021). Association of oral health factors related to oral function with mortality in older Japanese. Gerodontology.

[11] Vogtmann E., Etemadi A., Kamangar F. (2017). Oral health and mortality in the Golestan cohort study. Int J Epidemiol.

[12] Abnet C.C., Qiao Y.L., Dawsey S.M., Dong Z.W., Taylor P.R., Mark SD. (2005). Tooth loss is associated with increased risk of total death and death from upper gastrointestinal cancer, heart disease, and stroke in a Chinese population-based cohort. Int J Epidemiol.

[13] Aida J., Kondo K., Yamamoto T. (2011). Oral health and cancer, cardiovascular, and respiratory mortality of Japanese. J Dent Res.

[14] Kotronia E., Brown H., Papacosta A.O. (2021). Oral health and all-cause, cardiovascular disease, and respiratory mortality in older people in the UK and USA. Sci Rep.

[15] Holmlund A., Holm G., Lind L. (2010). Number of teeth as a predictor of cardiovascular mortality in a cohort of 7,674 subjects followed for 12 years. J Periodontol.

[16] Adolph M., Darnaud C., Thomas F. (2017). Oral health in relation to all-cause mortality: the IPC cohort study. Sci Rep.

[17] Iinuma T., Arai Y., Takayama M. (2016). Association between maximum occlusal force and 3-year all-cause mortality in community-dwelling elderly people. BMC Oral Health.

[18] Maekawa K., Ikeuchi T., Shinkai S. (2020). Number of functional teeth more strongly predicts all-cause mortality than number of present teeth in Japanese older adults. Geriatr Gerontol Int.

[19] Hirotomi T., Yoshihara A., Ogawa H., Ito K., Igarashi A., Miyazaki H. (2006). A preliminary study on the relationship between stimulated saliva and periodontal conditions in community-dwelling elderly people. J Dent.

[20] Gotoh S., Watanabe Y., Takeda M., Tomitsuka S. (2002). The chewing gum test: a sialometric evaluation for diagnosing Sjögren's syndrome. J Jpn Soc Oral Mucous Memb.

[21] Fried L.P., Tangen C.M., Walston J. (2001). Frailty in older adults: evidence for a phenotype. J Gerontol A Biol Sci Med Sci.

[22] Sasaki S. (2004). Research for evaluation methods of nutrition and dietary lifestyle programs held on healthy Japan 21: summary report.

[23] Yamaga T., Ogawa H., Miyazaki H. (2019). Influence of occlusal deterioration considering prosthetics on subsequent all-cause mortality in a Japanese elderly independent population. Gerodontology.

[24] Axelsson P., Paulartder J., Lindhe J. (1998). Relationship between smoking and dental status in 35-, 50-, 65-, and 75-year-old individuals. J Clin Periodontol.

[25] Leite F.R., Nascimento G.G., Scheutz F., López R. (2018). Effect of smoking on periodontitis: a systematic review and meta-regression. Am J Prev Med.

[26] Hosoda T.K., Youichi Morita H (2002). Study on association of tooth loss with lifestyle and health condition. J Yonago Med Assoc.

[27] Maeda Y., Idoji S., Nishida K. (1996). Relation between occlusal support and masticatory efficiency, occlusal force-results from survey on the masticatory functions among three facilities in Osaka-(Translated from Japanese). J Jpn Prosthodont Soc.

[28] Österberg T., Tsuga K., Rothenberg E., Carlsson G.E., Steen B. (2002). Masticatory ability in 80-year-old subjects and its relation to intake of energy, nutrients and food items. Gerodontology.

[29] Ikebe K., Nokubi T., Morii K., Kashiwagi J., Furuya M. (2005). Association of bite force with ageing and occlusal support in older adults. J Dent.

[30] Appollonio I., Carabellese C., Frattola A., Trabucchi M. (1997). Influence of dental status on dietary intake and survival in community-dwelling elderly subjects. Age Ageing.

[31] Yoshida M., Kikutani T., Yoshikawa M., Tsuga K., Kimura M., Akagawa Y. (2011). Correlation between dental and nutritional status in community-dwelling elderly Japanese. Geriatr Gerontol Int.

[32] Inomata C., Ikebe K., Kagawa R. (2014). Significance of occlusal force for dietary fibre and vitamin intakes in independently living 70-year-old Japanese: from SONIC study. J Dent.

[33] Pandey D.K., Shekelle R., Selwyn B.J., Tangney C., Stamler J. (1994). Dietary vitamin C and β-carotene and risk of death in middle-aged men. Am J Epidemiol.

[34] Zhao L.G., Shu X.O., Li H.L. (2017). Dietary antioxidant vitamins intake and mortality: a report from two cohort studies of Chinese adults in Shanghai. J Epidemiol.

[35] Kubota Y., Iso H., Date C. (2011). Dietary intakes of antioxidant vitamins and mortality from cardiovascular disease: the Japan Collaborative Cohort Study (JACC) study. Stroke.

[36] Kushi L.H., Folsom A.R., Prineas R.J., Mink P.J., Wu Y., Bostick RM. (1996). Dietary antioxidant vitamins and death from coronary heart disease in postmenopausal women. N Engl J Med.

[37] Abe K. (2021). Update on human research on antioxidant vitamins-how antioxidant vitamins work against oxidative stress-(Translated from Japanese). Chem Biol.

[38] Geraci A., Calvani R., Ferri E., Marzetti E., Arosio B., Cesari M. (2021). Sarcopenia and menopause: the role of estradiol. Front Endocrinol.

[39] Li M., Yan X., Wu R. (2025). Prevalence and diagnostic strategies for sarcopenia in menopausal and non-menopausal women: a cross-sectional comparative study. Int J Womens Health.

[40] Alimoradi Z., Jafari E., Roshandel Z., Potenza M.N., Lin C.Y., Pakpour AH. (2024). Meta-analysis with systematic review to synthesize associations between oral health related quality of life and anxiety and depression. BDJ Open.

[41] Hajek A., Kretzler B., König HH. (2022). Oral health, loneliness and social isolation. a systematic review and meta-analysis. J Nutr Health Aging.

[42] Beniusiene A., Kontautiene V., Strukcinskiene B., Grigoliene R., Martisauskiene D., Jurgaitis J. (2024). Depression, anxiety, and stress symptoms (DASS-21) in elderly women in association with health status (SHSQ-25): a cross-sectional study. Healthcare.

[43] Menotti A., Mulder I., Nissinen A. (2001). Cardiovascular risk factors and 10-year all-cause mortality in elderly European male populations - The FINE study. Eur Heart J.

[44] Menotti A., Mulder I., Nissinen A., Giampaoli S., Feskens E.J., Kromhout D. (2001). Prevalence of morbidity and multimorbidity in elderly male populations and their impact on 10-year all-cause mortality: the FINE study (Finland, Italy, Netherlands, Elderly). J Clin Epidemiol.

[45] Romandini M., Baima G., Antonoglou G., Bueno J., Figuero E., Sanz M. (2021). Periodontitis, edentulism, and risk of mortality: a systematic review with meta-analyses. J Dent Res.

[46] Iwasaki M., Borgnakke W.S., Yoshihara A. (2018). Hyposalivation and 10-year all-cause mortality in an elderly Japanese population. Gerodontology.

[47] Vital Statistics (Japanese). In: Ministry of Health LaW, ed.

[48] Yoshida M., Morikawa H., Yoshikawa M., Tsuga K., Akagawa Y. (2005). Eight-year mortality associated with dental occlusion and denture use in community-dwelling elderly persons. Gerodontology.

[49] Kobayashi S., Yuan X., Sasaki S. (2019). Relative validity of brief-type self-administered diet history questionnaire among very old Japanese aged 80 years or older. Pub Health Nutr.

